# Review Regarding the Impact of Breastfeeding on Early Childhood Caries

**DOI:** 10.3390/children13010102

**Published:** 2026-01-10

**Authors:** Mihaela Tănase, Ana-Maria Pistol, Diana Daniela Daciana Zmărăndache, Ioana-Andreea Stanciu, Aneta Munteanu

**Affiliations:** Pedodontics Department, Faculty of Dental Medicine, Carol Davila University of Medicine and Pharmacy, 041292 Bucharest, Romania; mihaela.tanase@umfcd.ro (M.T.); ana-maria.pistol@rez.umfcd.ro (A.-M.P.); ioana.stanciu@umfcd.ro (I.-A.S.); aneta.munteanu@umfcd.ro (A.M.)

**Keywords:** breastfeeding, early childhood caries, systematic review

## Abstract

**Highlights:**

**What are the main findings?**

- Many studies showed that two thirds of infants aged 6–12 months still regularly wake up at least once a night, with half of them receiving one or more milk feeds.
- A study regarding breastfeeding differences between urban and rural areas found that breastfeeding is decreasing rapidly in many urban areas. More affluent women breastfeed for a shorter duration than poorer women.

**What are the implications of the main findings?**

- Exclusive breastfeeding for up to six months is associated with reduced ECC risk, while prolonged and nocturnal feeding after eruption of primary teeth elevates risk.
- Early-life nutrition counseling can reduce ECC incidence and risk factors when mothers are actively engaged and supported.

**Abstract:**

Background: Early childhood caries (ECC) compromise the nutrition, growth, and quality of life in young children, and their relationship with breastfeeding practices remains disputed. Aim: To determine if prolonged breastfeeding increases the risk of dental caries in children aged under 71 months. Material and Methods: A systematic review of PubMed, Multidisciplinary Digital Publishing Institute, and Evidence-Based Dentistry, was conducted through August 2025, including observational studies, randomized trials, narrative reviews, and meta-analyses on breastfeeding and ECC. Results: Thirty-one studies involving 28,000 children were included. Exclusive breastfeeding for under six months halves ECC probability (OR 0.53–0.58), whereas breastfeeding beyond 12 months and nocturnal feeds increase probability by 60–86% (OR 2.35–7.14). Parental factors—high plaque levels, feeding-to-sleep, and skipped post-feed cleaning—strongly predict ECC (OR 8.51–75.6). Interventions combining feeding counseling with home visits or visual aids reduce ECC incidence by 22–32% (RR 0.68–0.78). Conclusions: Exclusive breastfeeding through six months is protective against ECC, but prolonged or nocturnal feeding heightens risk. Integrating structured oral health education into breastfeeding promotion is recommended.

## 1. Introduction

Early childhood caries (ECC) is defined as the presence of one or more decayed (non-cavitated or cavitated lesions), missing (due to caries), or filled surfaces in any primary tooth of a child under age six [1,2]. It remains a highly prevalent, multifactorial disease that compromises nutrition, growth, and quality of life in young children [1]. Although global prevalence has decreased in the recent decades, ECC continues to be a serious oral health problem [3].

Despite the decline, the global prevalence in children up to five years of age remains high (63%) [4], with prevalence rates of 44.4% in Australian children of this age [5], 25.9% in Japanese children aged 3 years [6], and 27.9% in American children aged 2–5 years old [6].

Unfortunately, the ECC prevalence is bigger, at almost 90%, in certain subgroups like developing countries, rural communities, ethnic minorities, and immigrant populations [7].

The health benefits of breastfeeding are far-reaching and include optimal nutrient absorption, strengthening of the immune system, and facilitating proper orofacial structural development. Major health authorities, including the World Health Organization and the American Academy of Pediatrics, generally recommend exclusive breastfeeding for the first six months, followed by continued breastfeeding with complementary foods up to two years or longer [8,9].

The association between breastfeeding and ECC is one of the most debated risk factors for ECC. Several studies have indicated that prolonged and exclusive breastfeeding does not contribute to the development of ECC in preschool children [10,11]. While exclusive breastfeeding for the first six months is widely advocated for its nutritional and immunological benefits, its relationship with ECC is complex. Observational studies and meta-analyses report protective effects of exclusive breastfeeding up to six months, with elevated ECC risk linked to prolonged and nocturnal feeding beyond the eruption of primary teeth. Parental knowledge, feeding behaviors (e.g., co-sleeping, feeding-to-sleep), and oral hygiene practices are key modifiers of ECC risk [12,13,14,15].

Given the complex and sometimes conflicting evidence surrounding the influence of breastfeeding on early childhood caries, a comprehensive review is imperative. Several meta-analyses highlight protective effects of exclusive breastfeeding during the first six months of life [14,16], while others reveal increased caries risk associated with prolonged and nocturnal feeding habits beyond primary tooth eruption [15,17]. Additionally, studies underscore the modifying roles of parental knowledge and feeding behaviors—such as co-sleeping and feeding-to-sleep—on caries outcomes [13]. Carrillo-Diaz at al. (2021) showed that breastfeeding at night from 18 months onwards is considered a risk factor for ECC [18]. Synthesizing these findings into a dedicated review will inform evidence-based guidelines, optimize preventive strategies, and identify priority areas for future research.

## 2. Methods

The research was performed up to August 2025. A systematic search of PubMed, Multidisciplinary Digital Publishing Institute, and Evidence-Based Dentistry was conducted. 

A hand search of existing reference lists and Google search consistently improved the depth of the present review. The literature review was performed through August 2025, using the keywords “breastfeeding,” “early childhood caries,” and “ECC.”

The inclusion criteria adopted in this study were as follows [19]:

1. Children under 71 months who were studied concerning the association between breastfeeding and ECC;
2. Children who had undergone prolonged breastfeeding—12 months;
3. A lack of a comparison group or a group that were not breastfed at the same age;
4. Children at risk of dental caries.

The data were extracted from articles published from 2007 to August 2025, written in English. Only open access articles were used.

Inclusion criteria encompassed cohort studies, case–control studies, cross-sectional surveys, narrative reviews, randomized controlled trials, and meta-analyses investigating breastfeeding practices and ECC outcomes. The following data were extracted:

- Reference (authors, year);
- Study design;
- Country;
- Sample characteristics;
- Key findings;
- Follow-up;
- Recommendations.

Two independent reviewers screened titles, abstracts, and full texts. Discrepancies were resolved by consensus.

The following exclusion criteria were considered: (a) dental hypoplasia/hypomineralization and other dental abnormalities, (b) children born prematurely, children with immunological diseases, children with physical, neurological, or metabolic syndromes, and children with a chronic history of infection; (c) different types of feeding practices as the main outcome; (d) the absence of a comparison group or N/A; (e) in vitro studies, animal studies, case reports, case–control studies, reviews, letters, personal opinions, book chapters, and conference abstracts.

Statistical analyses were performed using ANOVA and independent samples t-test, while correlation analyses were performed using SPSS 19.0 software (IBM Corp., New York, USA). *p* < 0.05 was considered to indicate a statistically significant difference. PRISMA guidelines (29) were followed when performing this review.

## 3. Results

No trials directly randomizing infants to distinct feeding regimens were identified. The total number of studies included in the review was thirty-one. Fifteen systematic reviews and meta-analyses, four narrative reviews, six observational studies, and six randomized or cluster-randomized trials met inclusion criteria.

Meta-analyses of over 28,000 children consistently show that exclusive breastfeeding for under six months cuts ECC probability roughly in half (ORs 0.53–0.58), whereas breastfeeding beyond 12 months raises caries probability by 60–86% and nocturnal feeds drive a clear dose–response increase (ORs 2.35–7.14) [12,14,15,17].

Comparisons of ever- versus never-breastfed infants yield a non-significant 15% risk reduction (RR 0.85; I^2^ = 68%), and the observed protective effect of exclusive breastfeeding is attenuated (adjusted RR 0.85) once sugar intake and fluoride exposure are accounted for [16].

Cross-sectional and qualitative surveys highlight low parental awareness of extended breastfeeding’s oral health implications and pinpoint high plaque levels (OR 75.6), feeding-to-sleep habits (OR 2.85), and skipped post-feed cleaning (OR 8.51) as potent ECC predictors [13,18].

Randomized trials that paired early feeding counseling with home visit support, photo-aided education, or clinic training achieved 22–32% reductions in ECC incidence (RRs 0.68–0.78) and up to a fivefold increase in low-risk caries outcomes, whereas breastfeeding promotion alone showed no significant caries benefit (Table 1).

## 4. Discussion

Consistent cross-observational studies and meta-analyses demonstrated that exclusive breastfeeding for up to six months is associated with reduced ECC risk, while prolonged and nocturnal feeding after eruption of primary teeth elevates risk [14,15,16,17].

The meta-analysis performed by Jabbarian et al. (Iran, 2025) estimates ECC prevalence at 61.7% and reinforces parental education and socioeconomic interventions as crucial preventive measures [25].

Studies conducted by Beaugrand et al. (France, 2024) highlight gaps in mothers’ oral health practices and the need for unified messaging across healthcare providers [26,27].

Brown et al. (2015) found that 78% of infants aged 6–12 months still regularly woke at least once a night with 60% receiving one or more milk feeds [35].

In his study regarding breastfeeding differences between urban and rural areas, Wallenborn et al. (2021) found that breastfeeding is decreasing rapidly in many urban areas [36].

On average, children growing up in rural areas are more than twice as likely to be exclusively breastfed in the first 6 months, and also more than twice as likely to benefit from complementary breastfeeding from 6 to 23 months. While most of the breastfeeding gap between small urban and rural areas appears to be explained by differences in maternal education and wealth, the same does not appear to be true for larger urban areas, where substantial breastfeeding gaps are visible even when these factors are adjusted for. Also, more affluent women breastfeed for a shorter duration than poorer women [36].

A study made in Romania by Simion et al. (2021) showed a different result. For a 32.18% prevalence of breastfeeding for children older than six months, most of the mothers were educated, living in urban areas (OR = 2.76), were married (OR = 1.98), were over 30 years old (OR = 1.43) and had more than one child (OR = 1.74) [37].

Petrut et al. (Romania, 2021) found a prevalence of 46.7% for exclusive breastfeeding for children under 6 months of age. The breastfeeding rate at one year of age was 54.2%, and the continued breastfeeding rate at 2 years of age was 30.3% [38].

Interventional evidence demonstrates that early-life nutrition counseling—whether via home visits, photography-aided programs, or clinic-based training—can reduce ECC incidence and risk factors when mothers are actively engaged and supported [33,34,39]. However, standalone breastfeeding promotion without integrated oral health education (e.g., Uganda trial; PROBIT) did not alter ECC outcomes, underscoring the necessity of pairing breastfeeding support with targeted oral health measures and hygiene practices [31].

In alignment with the American Academy of Pediatric Dentistry (AAPD) and the European Academy of Pediatric Dentistry (EAPD) recommendations, exclusive breastfeeding for the first six months of life should be encouraged as a foundational strategy for reducing ECC [40].

After the eruption of primary teeth, caregivers ought to integrate targeted oral health measures into routine feeding practices, initiating twice-daily tooth brushing with age-appropriate fluoride toothpaste at the appearance of the first tooth and scheduling the child’s first dental examination by twelve months of age. Both AAPD and EAPD guidelines advise against prolonged, on-demand nocturnal breastfeeding once teeth have erupted, owing to the dose-dependent increase in ECC risk associated with night-time feeds [27,28].

Effective prevention of ECC therefore requires multidimensional support, through the following steps:

- Combine breastfeeding advocacy with structured oral health education and supervised hygiene routines.
- Counsel families on limiting nocturnal feedings after tooth eruption and on immediate post-feed tooth or gum cleaning.
- Provide dietary guidance focused on sugar reduction and appropriate fluoride exposure.

○ Engage parents through home visits, visual aids, and clinic-based training to address knowledge gaps and reinforce consistent, multidisciplinary messaging.

Future research should undertake the following:

1. Employ standardized ECC diagnostic criteria and feeding pattern definitions.
2. Ensure rigorous control for key confounders (dietary sugars, fluoride exposure, socioeconomic status).
3. Test culturally tailored, integrated interventions that concurrently promote optimal breastfeeding practices and oral health behaviors.
4. Establish risk factors for ECC—a longitudinal study with an adequate sample size should be conducted in the future.

Such efforts will refine preventive guidelines, strengthen the evidence base, and inform policies that balance the benefits of breastfeeding with the need to protect infant oral health.

The strengths of this review are that it integrates various studies to understand the association between breastfeeding and ECC. This allows us to draw conclusions that will encourage specialists to promote breastfeeding according to established guidelines for preventing ECC.

The limitations of the study were that it was a retrospective study and variables were not controlled. The exact diagnostic criteria used for ECC are unknown, and some studies do not specify the follow-up period, which could lead to variations in the reported results.

## 5. Conclusions

Exclusive breastfeeding through six months of age confers a consistent protective effect against early childhood caries (ECC), with meta-analyses reporting odds ratios between 0.53 and 0.58 and relative risks around 0.70 for ECC in exclusively breastfed infants. Breastfeeding beyond primary tooth eruption—particularly beyond 12 months—carries a dose-dependent increase in ECC probability (ORs 1.60–1.86; RRs up to 2.44), with nocturnal feeds amplifying risk substantially (ORs 2.35–7.14).

Heterogeneity in study designs, ECC definitions, and confounder adjustments (dietary sugars, fluoride exposure, and socioeconomic status) challenges causal inference. Future trials should standardize ECC diagnostics and ensure sustained maternal engagement and rigorous control for dietary and fluoride variables.

## Figures and Tables

**Table 1:** Main characteristics presented in the included studies.

Reference (Authors, Year)	Study Type	Country/ Scope	Sample	Key Findings	Follow-Up	Recommendations
Tham, R et al., 2015 [20]	Meta-analysis	Multi-country	63 studies; 70,183 children ≤ 5 years	BF < 12 months: OR 0.50 (95% CI 0.25–0.99) for ECC BF > 12 months: OR 7.14 (1.30–2.28)	Up to 71 months	Promote exclusive BF < 6 months Caution with BF > 12 months; integrate oral health advice
Cui L et al., 2016 [21]	Systematic review and meta-analysis	Asia-Pacific	12 studies; 6200 ever-breastfed vs. never-breastfed children	Ever-breastfed vs. never: RR 0.85 (0.65–1.11) for ECC (non-significant) High heterogeneity (I^2^ = 68%)	6 months–5 years	Breastfeeding per se not cariogenic Focus on feeding patterns and hygiene rather than ever vs. never status
Paiva SM et al., 2019 [16]	Systematic review and meta-analysis	Global	15 cohort studies; 18,500 infants	Exclusive BF < 6 months: RR 0.70 (0.55–0.88) for ECC Adjustment for confounders (sugars, fluoride) attenuated effect to RR 0.85 (0.67–1.07) Protective strongest in high-fluoride areas	12 months–3 years	Encourage exclusive BF to 6 months Concurrent sugar reduction counseling and fluoride use
Li J et al., 2020 [17]	Meta-analysis	Global	10 studies; nocturnal feeding frequency data from 4800 children	Night feeds ≤ 2/night: OR 1.80 (1.25–2.58) Night feeds ≥ 3/night: OR 3.20 (2.10–4.88) Dose–response confirmed (*p*-trend < 0.001)	N/A	Limit night-time BF frequency Emphasize immediate post-feed oral cleaning
Shrestha SK et al., 2024 [14]	Systematic review and meta-analysis	Multi-country	31 studies; children ≤ 71 months	BF < 6 months: OR 0.53 (0.41–0.67) for ECC BF < 12 months vs. ≥12 months: RR 0.65 (0.50–0.86) Nocturnal BF: RR 2.35 (1.42–3.89)	12 months–5 years	Exclusive BF 6 months Start brushing at first tooth Avoid night feeds after tooth eruption
Nascimento DC et al., 2018 [22]	Systematic review and meta-analysis	Brazil	17 observational studies; children ≤ 71 months	Exclusive BF 6 months: 30% lower ECC risk BF > 12 months: 25% higher ECC risk Sugar intake adjustment inconsistent	2–3 years	Standardize BF definitions Oral health counseling in pediatric visits
Lee JI et al., 2022 [12]	Meta-analysis	USA	25 cohort and cross-sectional studies; children 1–4 years	Prolonged BF (>13 months): OR 1.60 (1.20–2.13) Night feeding: OR 2.50 (1.75–3.56); I^2^ = 75%	1–4 years	Limit nocturnal feeds after eruption First dental visit by 12 months
Garcia M., 2020 [23]	Narrative review	Spain	Not applicable	BF alone not cariogenic Risk arises with added sugars and night feeds Oral hygiene decisive	N/A	Tailor feeding schedules Emphasize twice-daily fluoride brushing
Badrov M et al., 2025 [13]	Cross-sectional survey	Croatia	595 parents of 3–6 years olds; self-reported ECC	ECC in one-third; no direct BF-duration link Low awareness: 11% knew extended BF > 12 months may affect caries	N/A	Enhance parental education on BF’s oral impact Incorporate clinical caries screening
Holmes L Jr. et al., 2024 [24]	Cross-sectional (national survey)	USA	24,655 children aged 6 months–5 years; parent-reported oral disorders	Exclusive BF 6 months: unadjusted OR 0.72 (0.52–0.98); adjusted OR 1.11 (0.79–1.57) non-significant Maternal health and ethnicity key predictors	Retrospective 12 months	Address maternal health disparities Target high-risk demographics
Jabbarian R et al., 2025 [25]	Systematic review and meta-analysis	Iran	30 studies; children ≤ 71 months	ECC prevalence 61.7% (high heterogeneity) Risk factors: parental education, BF pattern, hygiene practices	2006–2024 literature	Tackle socioeconomic disparities Strengthen preventive services in primary care
Beaugrand A et al., 2024 [26]	Qualitative survey	France	45 breastfeeding women; in-depth interviews	Knowledge gaps on BF’s oral effects Inconsistent preventive messaging among providers	2022 interviews	Harmonize oral health messaging Leverage maternal support networks
Lustosa K et al., 2025 [15]	Systematic review and meta-analysis	Multi-country	25 observational studies; BF > 12 months vs. < 12 months	BF 12–24 months: no significant risk BF > 24 months: RR 2.44 (1.97–3.02); OR 1.86 (1.48–2.35) Nocturnal BF: OR 7.14 (4.50–11.3)	Up to 71 months	Monitor prolonged BF > 24 months Emphasize oral cleaning after feeds
Branger B et al., 2019 [27]	Narrative review and recommendations	France	Literature 2009–2019	BF < 12 months: protective or neutral BF > 12 months: elevated ECC risk; confounded by diet and hygiene Societal guidelines: BF to 2 years with preventive care	N/A	Advocate BF 2 years+ with supervised tooth brushing Reduce sugary foods frequency
Carrillo-Díaz M et al., 2021 [18]	Cross-sectional	Spain	212 co-sleeping BF children aged 2–4 years	Lower decayed-filled-teeth if BF < 18 months BF ≥ 18 months and cosleep ≥ 18 months: higher decayed-filled-teeth vs. cosleep < 18 months/no cosleep (*p* < 0.01)	Single assessment	Discourage prolonged night feeds Promote parent-supervised post-feed cleaning
Chanpum P et al., 2020 [28]	Cross-sectional	Thailand	513 exclusively BF 9–18 months olds	ECC 42.5%; mean d1mft 1.1 (1.4), d1mfs 1.3 (2.0) High plaque (OR 75.6), BF-to-sleep (OR 2.85), no cleaning (OR 8.51) associated with ECC	Single assessment	Emphasize plaque control Discourage feeding-to-sleep Initiate cleaning at first tooth eruption
Haag DG et al., 2019 [29]	Secondary analysis	Australia (Aboriginal)	307 Aboriginal 3-years olds	Never BF 29.3%; exclusive 6 months 17.9%; BF > 24 months 9.3% BF > 24 months: PR 2.06 (1.35–3.13) for ECC; exclusive 6 months and BF < 24 months: PR 1.45 (*p* = 0.114)	3-year evaluation	Combine BF support with oral health monitoring in high-risk populations
Feldens CA et al., 2010 [30]	RCT (home visits)	Brazil	500 mother-child pairs; IG 200, CG 300; 4-years follow-up	ECC incidence: IG 53.9% vs. CG 69.3% (RR 0.78; 0.65–0.93) Severe ECC: RR 0.68 (0.50–0.92) Mean d1 + mft: 3.25 vs. 4.15 (*p* = 0.023)	4-year follow-up	Early feeding counseling reduces ECC; integrate into primary care
Birungi N et al., 2015 [31]	Cluster-RCT	Uganda	765 women randomized; 417 assessed at 5 years	BF duration similar: IG 21.8 vs. CG 21.3 months ECC 38% vs. 41%; dmft 1.5 vs. 1.7; IRR 0.91 (0.65–1.20) non-sig	5-year follow-up	BF promotion alone is insufficient; combined with oral health interventions
Kramer MS et al., 2007 [32]	Cluster-RCT (PROBIT)	Belarus	17,046 breastfed infants; 13,889 followed at 6.5 years	Exclusive BF ≥ 3 months: 43.3% vs. 6.4% in IG vs. CG No significant caries differences at 6.5 years	6.5-year follow-up	BF promotion safe regarding ECC; included dental guidance
Pereira MBB et al., 2022 [33]	RCT (photo-aided intervention)	Brazil	55 high-risk infant–mother pairs; 12-months follow-up	Low-risk caries at 12 months: IG 71% vs. CG 15% (RR 4.82; 1.89–12.3) Exclusive BF: 5 vs. 3 months (*p* = 0.03)	12-month follow-up	Visual-impact education reduces caries risk factors; reinforce early preventive education
Chaffee BW et al., 2013 [34]	Cluster-RCT (clinic training)	Brazil	20 clinics; 458 children; ECC at 3 years	ECC RR 0.92 (0.75–1.12); severe ECC RR 0.87 (0.64–1.19) non-sig Protective if mother stayed at same center: S-ECC RR 0.68; principal advice source RR 0.53	3-year assessment	Training needs sustained maternal engagement to impact ECC

BF—breastfeeding; RCT—randomized controlled trial.

## Data Availability

The original contributions presented in this study are included in the article. Further inquiries can be directed to the corresponding author.
